# Essential psychiatric medicines: wrong selection, high consumption and social problems

**DOI:** 10.1186/s12889-015-2589-1

**Published:** 2016-01-20

**Authors:** Izabela Fulone, Silvio Barberato-Filho, Michele Félix dos Santos, Carolina de Lima Rossi, Gordon Guyatt, Luciane Cruz Lopes

**Affiliations:** 1 Pharmaceutical Sciences Master’s Course, University of Sorocaba, UNISO, Rodovia Raposo Tavares, KM 92,5 -Sorocaba, São Paulo, 18023-000 Brazil; 2 Clinical Pharmacology Graduate Course, University of São Francisco, USF, Campinas, Brazil; 3 Department of Clinical Epidemiology and Biostatistics, McMaster University, Hamilton, Canada

**Keywords:** Antidepressants agents, Benzodiazepines, Drug utilization, Drugs, Essential

## Abstract

**Background:**

The World Health Organization Essential Medicines List (WHO-LIST) and national essential medicines lists differ because many countries face significant challenges, such as product availability, cost, product quality and epidemiological disease profiles. In Brazil, governments pay for drugs that are included on the federal, state and municipal government (REMUME) lists. The extent to which municipal lists differ from state and national lists and from the WHO-LIST is unclear. We investigate the use of the WHO-LISTas a tool with which to evaluate the selection process for the essential psychiatric medicines in the public system coverage list of Brazilian communities (cities) and the use of the target drugs.

**Methods:**

Municipal health secretaries were interviewed regarding the selection process for REMUMEs and the antidepressants and benzodiazepines included in REMUMEs and reference lists. We calculated the use of REMUME drugs that appeared or did not appear on reference lists according to the defined daily dose (DDD) per 10,000 inhabitants.

**Results:**

Local physicians and pharmacists without specific training or explicit criteria developed the REMUMEs. Of the 13 drugs and 24 products (i.e., the different dosages of these 13 drugs) in the REMUMEs, 8 drugs and 10 products were included in at least one reference list and in one municipal list; 4 drugs and 6 products were included in at least one reference list but in none of the municipal lists; and 7 drugs and 8 products were included in at least one municipal list but in none of the reference lists. The antidepressants that appeared in at least one municipal list but in none of the reference lists represented 25.1 % (mean 60.9 DDD/10,000 inhabitants-day) of the usage. The benzodiazepines that appeared in at least one of the municipal lists but in none of the reference lists represented 14.7 % mean 18.5 DDD/10,000 inhabitants-day) of the usage.

**Conclusions:**

Brazilian cities have no rigorous processes for selecting the drugs that appear on their lists, and drugs that do not appear on the reference lists represent a significant proportion of antidepressant and benzodiazepine use, resulting in public health and social problems.

**Electronic supplementary material:**

The online version of this article (doi:10.1186/s12889-015-2589-1) contains supplementary material, which is available to authorized users.

## Background

Essential medicines are intended to be available in health systems at all times, in adequate amounts, in the appropriate dosages, with assured quality and adequate information, and at a price that the individual and the community can afford [1]. The World Health Organization Essential Medicines List (WHO-LIST) includes drugs that are judged to satisfy the prioritized health care needs of the population. The considerations in the selection of these drugs include public health relevance, evidence regarding efficacy and safety, and cost-effectiveness [2].

Since the 1970s, many low- and middle-income countries have started national programmes for essential drugs to promote the availability, accessibility, affordability, quality and rational use of medicines [3]. The WHO-LIST serves as a guide for the development of national essential medicines lists, aguide that can be modified according to the national context –for instance, according to the prevalent diseases in a particular country [4].

In 1964 –prior to the first WHO-LIST, which was developed in 1977– Brazil developed its first list of essential medicines (RENAME). Despite this pioneering initiative, the discontinuity of public policy in subsequent decades and long periods in which the RENAME list was not revised may have delayed consolidation of the concept of essential medicines among managers, health professionals and users [5]. The review of the RENAME list in 2010 addressed a number of medicines that are needed to treat and control diseases that are public health priority in Brazil [6].

Designating essential medicines is important in all areas, including psychiatric medication. Psychotropic medicines, such as antidepressants and benzodiazepines, are widely used to treat mental illness [7].

Since the 1960s, benzodiazepines have been extensively used to treat anxiety and sleeping problems [8, 9]. Although they have proven effective in treating anxiety disorders, concerns about drug abuse liability and physical dependence due to long-term use gave rise to ample controversy during the 1980s [10–12].

The annual incidence of long-term benzodiazepine use across North America and Europe is estimated to be between 0.4 and 6 %, with higher rates of chronic use in patients older than 65 years of age [13–16]. Studies have suggested widespread suboptimal use of benzodiazepines in Brazil [17, 18]. For example, in the town of Belo Horizonte, 95 % of elderly respondents reported the use of benzodiazepines. In addition, in a small town of 10,000 inhabitants in the State of São Paulo, 50 % of the population used benzodiazepines [19].

Concerns have also been raised regarding the use of antidepressants. In 2009, Brazilians consumed 3.5 tons of fluoxetine, with evidence of abuse and use for purposes other than depression [17, 20].

In Brazil, governments pay for drugs that are included on essential medicines lists. Such lists are produced not only by federal and state governments but also by municipal governments. The list committees recruit members with documented expertise and with explicit criteria to develop federal and state lists. However, it is unclear whether municipal lists require such vetting, and the extent to which municipal lists differ from state, national, and international lists is similarly ambiguous.

Accordingly, we studied the development process forMunicipal Essential Medicines Lists (REMUMEs) in three Brazilian communities, documented the extent to which these communities’ lists overlapped with the state and national lists and the WHO-LIST in terms of benzodiazepine and antidepressant use, and reportedthe extent of use of the drugs included on the municipal lists but on no other lists.

## Methods

The committee for clinical research at the University of Sorocaba approved this study on April 13, 2010, with protocol number 003/2010.

We conducted this researchin three Brazilian cities in the State of São Paulo (which we will designate as cities 1, 2, and 3). The cities had a total of 519,924 inhabitants [21], and the Human Development Index was approximately 0.8 [22].

The current study included antidepressants (N06A), classified according to the third level of the Anatomical Therapeutic Chemical (ATC)/Defined Daily Dose (DDD), and all benzodiazepine derivatives, as identified in ATC’s fourth level. We included benzodiazepine derivatives classified as anxiolytics (N05B), hypnotics and sedatives (N05C), and antiepileptic drugs (N03AE) [23].

The selected medicines in this study were classified according to the ATC system proposed by the World Health Organization (WHO). The use of the ATC system allows the standardization of drug groupings and comparisons of drug use between countries, regions, and other health care settings. In the ATC classification system, the active substances are divided into different groups according to the organ or system on which they act and their therapeutic, pharmacological and chemical properties.

The drugs are classified into groups at five different levels. The drugs are divided into fourteen main groups (1^st^ level) with pharmacological/therapeutic subgroups (2^nd^ level). The 3^rd^ and 4^th^ levels are chemical/pharmacological/therapeutic subgroups, and the 5^th^ level concerns the chemical substance. The 2^nd^, 3^rd^ and 4^th^ levels are often used to identify pharmacological subgroups when such subgroups are considered more appropriate than therapeutic or chemical subgroups [23].

The data on consumption were obtained from the databases of each local mental health department. The medicine consumption was calculated using the ATC/DDD methodology, recommended by the WHO, and was expressed according to the DDD per 10,000 inhabitants per day.

We used the city population (adult and paediatric populations) receiving assistance as the denominator for DDD. The estimated percentage of inhabitants who obtained drugs from the public health system in city 1 was approximately 75 % (36,670); in city 2, the estimated percentage was 60 % (259,088); and, in city 3, the estimated percentage was 70 % (60,543). These data were obtained from the municipal health secretaries.

To obtain each REMUME and to explore the process of selecting the essential medicines included on the REMUMEs, we interviewed the director of the Departments and Pharmaceutical Assistance of the Municipal Health Secretary in each municipality. The interview addressed the composition of the committee, the declaration of conflicts of interest, the criteria for the selection of drugs, and frequency of updates (see Additional file 1).

We used the WHO-LIST published in 2011 (WHO-LIST) [24], the national essential medicines list published in 2010 (RENAME) [6] and the State of São Paulo Essential Medicines Lists published in 2011 (RESME) [25] as reference lists. To ensure the comparability of municipal lists with reference lists, we used the reference lists in force at the same time.

## Results

The selection process for essential medicines in all three municipal health departments was very similar. We’ve summarized all answers from the survey in Table 1. Physicians and pharmacists participate in the decision-making process, though nurses do not. This group of health professionals is known as a Pharmacy and Therapeutics Committee in city 2, but not in cities 1 and 3. In each city, the health care professionals who participate in the selection process recommend particular medicines, but the health secretary makes the final decision. There is no provision for the declaration of conflicts of interest among members of the selection committee.

**Table 1:** Characteristics of the selection process foressential medicines in cities 1, 2, and 3

Items	City 1	City 2	City 3
Process			
Official designation of PTC^a^	N	Y	N
Annual review	N	N	N
Declaration of interest	N	N	N
Cost analysis is crucial to include medicines	Y	Y	Y
Final decision is made by Health secretary	Y	Y	Y
Criteria for drug selection			
Professional experience	Y	Y	Y
Presence on reference list	Y	Y	Y
Epidemiology of disease	Y	Y	Y
Source of information			
Pharmaceutical industry catalogue	N	N	N
Guidelines of reference list	N	N	N
Systematic review/synopsis of evidence	N	N	N
Committee			
Multidisciplinary members	N	N	N
Expertise in selection essential medicines	N	N	N
Need for improve the selection process^b^	Y	Y	Y
Need for training the PTC^b^	Y	Y	Y

*Y* yes, thischaracteristic is present, *N* no, thischaracteristic is not present

^a^ *PTC* Pharmacy and Therapeutic Committee

^b^Opinion from each Health Secretary of city

Professional experiences are used as sources of information during the selection process for essential medicines. Epidemiological data and reference lists (WHO-LIST, RENAME, RESME) can be used to make decision. Although the selection criteria include the drug’s efficacy, safety, financial impact and feasibility of use, they don’t consider data from systematic reviews, synopses of evidence or guidelines. The financial analysis involves the cost of the medicine, the cost of treatment and the medicine’s cost compared to that of other drugs. Cost is always an important consideration.

The REMUMEs are not reviewed annually. The health departments acquire and pay for medicines beyond those provided by the state and federal managers. The health secretary only accepts requests for the inclusion of medicines from health professionals, thus not accepting those from patients or non-professional organizations. When asked about what could be improved in the selection process, the health secretaries identified education in drug selection and access to data summaries that, because of their costs, perhaps could not be obtained at that time.

Table 2 presents the antidepressants and benzodiazepines and the drug dosages on the REMUME and the worldwide (WHO-LIST), national (RENAME) and state (RESME) essential medicines lists. We identified 13 drugs and 24 products (i.e., the different dosages of these 13 drugs). Of the relevant drugs/products, 8 drugs and 10 products were included in at least one reference list and one municipallist; 4 drugs and 6 products were included in at least one reference list but in none of the municipal lists; and 7 drugs and 8 products were included in at least one municipal list but in none of the reference lists.

**Table 2:** Presence of antidepressants and benzodiazepines in all essential medicines lists (WHO-LIST, National, State and Municipal)

	World list	National list	State list-SP	REMUMEs					
ATC code	Generic name	Dosage	Pharmaceutical form	WHO-LIST	RENAME	RESME-SP	CITY 1	CITY 2	CITY 3
				2011	2010	2011	2009	2010	2010
Antidepressants Total									
N06AA	Tricyclic Antidepressants								
N06AA09	amitriptyline	25 mg	tablet	Y	Y	Y	Y	Y	Y
N06AA04	clomipramine	10 mg	tablet	Y	Y	Y	Y	Y	N
N06AA04	clomipramine	25 mg	tablet	Y	Y	Y	Y	Y	Y
N06AA10	nortriptyline	10 mg	capsule	N	Y	N	N	N	N
N06AA10	nortriptyline	25 mg	capsule	N	Y	Y	Y	Y	Y
N06AA10	nortriptyline	50 mg	capsule	N	Y	N	N	N	N
N06AA10	nortriptyline	75 mg	capsule	N	Y	N	N	N	N
N06AA02	imipramine	25 mg	tablet	N	N	N	Y	Y	Y
N06AB	Selective Serotonin Reuptake Inhibitors								
N06AB03	fluoxetine	20 mg	tablet	Y	Y	Y	Y	Y	Y
N06AB03	fluoxetine	20 mg/5 mL	oral solution	N	N	N	Y	Y	N
N06AB06	sertraline	50 mg	tablet	N	N	N	Y	Y	Y
N06AB04	citalopram	20 mg	tablet	N	N	N	Y	N	N
N06AX	Other Antidepressants								
N06AX12	bupropion	150 mg	tablet	N	Y	N	Y	N	N
N03AE/N05BA/N05CD Benzodiazepines									
N05BA09	clobazam	10 mg	tablet	N	N	N	Y	N	N
N03AE01	clonazepam	2.5 mg/mL	oral solution	N	Y	Y	N	Y	N
N03AE01	clonazepam	0.5 mg	tablet	N	N	N	N	N	Y
N03AE01	clonazepam	2 mg	tablet	N	N	N	Y	N	Y
N05BA01	diazepam	5 mg	tablet	Y	Y	Y	N	N	N
N05BA01	diazepam	10 mg	tablet	Y	N	N	Y	Y	Y
N05BA01	diazepam	2 mg/5 mL	oral solution	Y	N	N	N	N	N
N05BA01	diazepam	5 mg/mL	injection	Y	Y	N	N	Y	N
N05CD08	midazolam	5 mg/mL	injection	Y	N	N	N	Y	N
N05CD08	midazolam	2 mg/mL	oral solution	Y	Y	N	N	N	N
N05CD02	nitrazepam	5 mg	tablet	N	N	N	Y	Y	Y
Total	*n* = 24 (100)			10 (41.6)	13 (54.2)	7 (29.1)	14 (58.3)	13 (54.2)	10 (41.6)

*Y* yes, the medicine is present in the list, *N* no, the medicine is not present in the list

*REMUME* Municipal Essential Medicines List, *WHO*-*LIST* World Health Organization Essential Medicines List

*RENAME* National Essential Medicines List, *RESME*-*SP* State of São Paulo Essential Medicines List

Table 3 shows the antidepressant and benzodiazepine consumption in DDD/10,000 inhabitants. Of the antidepressants, selective serotonin reuptake inhibitors (SSRIs), particularly fluoxetine, showed the highest use. The most-used benzodiazepine was diazepam. Of the antidepressants, the drugs that appeared on a municipal list (imipramine + sertraline + citalopram) but not on any of the reference lists represented 25.1 % (60.9 mean DDD-10,000 inhabitants-day) of the usage. The benzodiazepine drugs that appeared on a municipal list (nitrazepam + clobazam) but not on any of the reference lists represented 14.7 % (18.1 mean DDD-10,000 inhabitants-day) of the usage.

**Table 3:** Comparison of consumption in DDD-10,000 inhabitants in the three Brazilian cities studied

ATC code	Generic names	Presence in WHO/RENAME and/or RESME	City 1	City 2	City 3	Mean + Std Dev(%)
			2009	2010	2010	
	Antidepressants Total	-	242.6	229.9	253.7	242.1 ± 11.9 (100 %)
N06AA	Tricyclic Antidepressants	-	40.3	37.4	35.6	37.7 ± 2.3 (15.5)
N06AA09	amitriptyline	Y	21.0	25.9	26.5	24.4 ± 3.0 (10.1)
N06AA04	clomipramine	Y	5.2	5.9	3.8	4.9 ± 1.1 (2.0)
N06AA10	nortriptyline	Y	0.6	2.3	1.6	1.5 ± 0.8 (0.6)
N06AA02	imipramine	N	13.5	3.1	3.6	6.7 ± 5.8 (2.7)
N06AB	SSRI Antidepressants ^a^	-	202.3	192.5	218.1	204.3 ± 12.9 (84.3)
N06AB03	fluoxetine	Y	152.9	136.1	161.1	150.0 ± 12.7 (61.9)
N06AB06	sertraline	N	49.3	56.4	56.9	54.2 ± 4.2 (22.4)
N06AB04	citalopram	N	0.1	NA	NA	-
N06AX	Other Antidepressants		1.9	-	-	-
N06AX12	bupropion	Y	1.9	NA	NA	-
N03AE/ N05BA/N05CD	Benzodiazepines Total	-	161.9	117.1	98.1	125.7 ± 32.7 (100 %)
N05CD08	midazolam	Y	NA	0.004	NA	-
N05CD02	nitrazepam	N	31.3	11.3	11.7	18.1 ± 11.4 (14.4)
N03AE01	clonazepam	Y	11.1	19.5	28.8	19.8 ± 8.8 (15.7)
N05BA09	clobazam	N	1.4	NA	NA	-
N05BA01	diazepam	Y	118.1	86.3	57.6	87.3 ± 30.2 (69.4)

*Y* yes, the medicine is present on the list, *N* no, the medicine is not present on the list, *NA* not available

^a^ *SSRI antidepressants* selective serotonin reuptake inhibitor antidepressants

## Discussion

We found that the REMUMEs in the three studied cities were developed by a small group of physicians and pharmacists who needs more formal training and expertise in the selection of essential medicines. In all three cites, the lists included antidepressants and benzodiazepines that were not included in the reference lists (i.e., the state, national and WHO lists). The antidepressants that did not appear on any of the reference lists represented 25.1 % of the antidepressant use, and the benzodiazepines that did not appear on the list represented 14.7 % of benzodiazepine use.

This study has a number of strengths. It is the first study to address how municipal essential medicines lists in Brazil differ from state, national and WHO essential medicines lists. We not only compared the drugs covered by the municipalities versus those covered by the reference lists but also explored the process used to determine the essential medicines list in the three cities under investigation and estimated the total use of the drugs that appeared and did not appear on the reference lists. The study thus provides a comprehensive picture of the potential problems of essential medicine designation and use in smaller Brazilian cities.

Our study has also limitations. The DDD, which we chose as the unit of measurement for drug use, does not necessarily reflect the prescribed daily dose – the patients may have used higher or lower doses than prescribed. However, the ATC/DDD standard methodology is the only system available that allows the comparison of the use of one drug versus another drug across different drugs.

We did not examine the reason for or appropriateness of the prescription of either antidepressants or benzodiazepines. Such an assessment would have required us to establish appropriateness criteria and to examine individual patient records.

The extent to which our results from three Brazilian cities are generalizable across the country is questionable. However, in these three cities, the selection processes and the drugs included on the municipal lists that did not appear on the reference lists were very similar. These consistencies suggest that the results may be widely generalizable.

The use of drugs that did not appear on the reference lists is potentially problematic. The WHO-LIST should guide the development of national lists, which in turn should guide the selection of drugs in states and municipalities [26]. The local committees that helped the health secretary in related decision making did not have explicit criteria to justify their discounting of the reference lists.

Because municipalities do not have selection criteria for products included or excluded from their lists, there is no way to discuss the relevance or quality of the selection process. However, the presence of these drugs on the municipal lists, which do not meet the criterion of essentiality, shows that such drugs were not selected based on scientific evidence or on the criterion of essentiality. The number of ‘me-too drugs’ in each list highlights this problem.

We found that, of the antidepressants, SSRIs, particularly fluoxetine, showed the highest levels ofuse. This finding makes sense given the greater tolerability of SSRIs and the correspondingly greater toxicity of tricyclic antidepressants, particularly in terms of their cardiotoxicity [27]. Effective marketing, particularly that associated with the launch of fluoxetine, may contribute to the high levels of SSRI use. The increasing use of SSRIs is consistent with trends in other countries [28–30].

The number of different types of benzodiazepines found in each municipality list and also the high levels of use suggest the wrong selection, the chronic use and potentially the overuse of the drugs. Such results show the extent of the problem, and they should be considered in planning interventions to rationalize the use of these drugs in Brazil, particularly through health programme planning.

It is important to note that the chronic use of benzodiazepines may result in dependence, tolerance, impairment to cognitive abilities, and memory problems [31], which may represent a major economic burden associated with benzodiazepine-related drug interactions, particularly in elderly persons [32]. Such overuse is widespread in other settings [17, 18, 33, 34].

The chronic use and overuse of benzodiazepines may reveal the social problems associated with such use. There is often an unquestioning belief in the biomedical model for treating mental illness, which focuses only on the drug and does not consider the health/disease/care processes embedded in particular contexts by social factors; there is a subjective and cultural dimension that will affect the way people use such drugs and formal health care services.

Discussing the socio-cultural dimensions of these issues could enhance prescribers’ and users’ understanding of the risks involved in chronic use and overuse.

This scenario reflects on the federal government. The municipalities’ failure to adhere to Brazil’s public health policy, represented by the wrong selection of medicines for inclusion on their lists, reinforces their prescription and inappropriate use.

To remedy these problems, smaller cities in Brazil should establish formal Pharmacy and Therapeutic Committees and should only include drugs that appear on the reference lists, particularly the WHO-LIST, on the municipal lists. Exceptions should be restricted to instances in which a compelling reason exists for a drug’s inclusion. Such situations are likely uncommon.

## Conclusions

The findings highlight the lack of rigorous criteria for determining which drugs to include in Brazilian cities’ essential medicines lists. Cities frequently include drugs that do not appear on the reference lists, and these drugs represent an appreciable proportion of use of antidepressants and benzodiazepines. The wrong selection of medicines introduces inappropriate drug use and social problems.

### Ethics approval

This project was approved by the committee for clinical research at the University of Sorocaba on April 13, 2010, with protocol number 003/2010.

## Additional file

Additional file 1:
**Tool used to assess the Pharmacy and Therapeutics Committees of the cities.** (DOCX 14 kb)

## Abbreviations

ATC: Anatomical therapeutic chemical
DDD: Defined daily doses
SSRIs: Selective serotonin reuptake inhibitors
PTC: Pharmacy and therapeutic committee
REMUME: Municipal essential medicines list
RENAME: National essential medicines list
RESME: State of Sao Paulo essential medicines list
WHO: World Health Organization
WHO-LIST: World Health Organization (WHO) essential medicines list
