# Supplementary material for: Essential psychiatric medicines: wrong selection, high consumption and social problems
Source: BMC Public Health. 2016 Jan 20;16:52. doi: 10.1186/s12889-015-2589-1 (PMC4719662; doi:10.1186/s12889-015-2589-1)
Supplement: Additional file 1: — Tool used to assess the Pharmacy and Therapeutics Committees of the cities. (DOCX 14 kb) [file 12889_2015_2589_MOESM1_ESM.docx]

**Additional file 1**–**Tool used to assess the Pharmacy and Therapeutics Committees of the cities** (adapted from Marques, 2006)^1^

**Guidance to fill:**

This questionnaire consists of essay and multiple choice questions.

The questions with options that are not mutually exclusive may have multiple alternative choices.

Responsible for filling:

Profession:

Role:

Date:

**1**. Is pharmaceutical assistance provided in the municipal health plan?

( ) Yes ( ) No

**2**. Does the Health Municipal department acquire medicines in addition to those provided by the state and federal manager?

( ) Yes ( ) No

**3**. If yes, which professionals are involved in the medicine selection to make the list of essential medicines?

( ) group/ professional from technical area

( ) group/ professional from the administrative area

( ) group/professional from the technical and administrative area

( ) a health secretary manager

( ) other Specify:

**4**. If it is a group of professionals, is this group characterized as a Pharmacy and Therapeutics Committee?

( ) Yes ( ) No

**5**. Which professionals are responsible for the selection of medicines in the Municipal Health department?

( ) physicians Specialties:

( ) pharmacists

( ) nurses

( ) dentists

( ) manager

( ) other To specify:

**6**. When are reviews conducted for inclusions or exclusions of medicines on the list?

( ) when needed

( ) in a period of time up to 1 year

( ) in a period of time 1 to 2 years

( ) in a period of time > 2 years

( ) other period To specify:

**7**. Do the group/professionals that make the medicine selection receive requests for inclusion or exclusion of medicines from the list?

( ) Yes ( ) No

**8**. If yes, who makes requests?

( ) health secretary professionals

( ) pharmaceutical industry

( ) users of health service

( ) organizations of the society

( ) other To specify:

**9**. Those who make requests should provide what type of information?

( ) anything

( ) indications of use

( ) therapeutic quality

( ) references from the literature

( ) other To specify:

**10** . Do Epidemiological data influence the process of medicine selection?

( ) Yes ( ) No

**11**. Which information sources are used in the medicine selection?

( ) professionals’ experience

( ) reference lists (WHO, RENAME, RESME)

( ) National Therapeutic Form (Brazil)

( ) guidelines of reference

( ) pharmaceutical industry catalogue

( ) systematic review/ synopses of evidence

( ) other To specify:

**12**. What parameters are used for the evaluation of medicines that will be included or excluded from the list?

( ) efficacy

( ) safety

( ) cost

( ) availability on the pharmaceutical market

( ) organization of services to incorporate the medicines

**13**. If the cost was marked on the previous question, which aspects of cost are analysed?

( ) cost of medicine

( ) cost of treatment

( ) estimate of the expenditure over a period of time

( ) cost compared to another medicine (cost-minimizing)

( ) other To specify:

**14**. The result of the cost analysis is crucial to include or not include a medicine on the list?

( ) always

( ) never

( ) sometimes

**15**. Does the group that makes the medicine selection in the municipality make the decision to include or exclude medicines from the list?

( ) Yes ( ) No

**16**. If yes, who is the responsible for the final decision?

( ) the manager of the group/ professionals

( ) the health secretary

( ) the municipal health council

( ) other To specify:

**17**. Is there a term for a declaration of conflicts of interest?

( ) Yes ( ) No

**18**. In your opinion, what can be improved in the medicine selection?

( ) nothing

( ) the information analysed about medicines

( ) the expertise of professionals involved in the selection process

( ) the disclosure of the criteria for the medicine selection

( ) adherence of the prescribers to the essential medicines list

( ) other To specify:
